# On Croup

**Published:** 1850-09

**Authors:** 


### ECLECTIC DEPARTMENT,

### AND SPIRIT OF THE MEDICAL PERIODICAL PRESS.

---

*On Croup.* By JOHN WARE, M. D., Prof. of the Theory and Practice of Medicine, Massachusetts Medical College.

We had intended to have condensed these articles by Prof. Ware, but, on looking them over for that purpose, we have been unable to bring our mind to strike out any part of them. We have, therefore, determined to insert them entire, notwithstanding they will occupy nearly, or quite all the Eclectic Department of this Number of the Journal. In our opinion, we could not select any contributions to practical medicine more interesting and valuable than these several papers. The final conclusions respecting the treatment of Croup, at which the author arrives, will be found to conflict directly with the views generally entertained by practical physicians. This, however, is no reason why the observations and reasoning upon which Prof. W. bases these conclusions should not be carefully and candidly considered.

The several articles have recently appeared in the Boston Medical and Surgical Journal. EDITOR BUFFALO MEDICAL JOURNAL.

I.—*Contributions to the History and Diagnosis of Croup.*—Read before the Boston Society for Medical Improvement, in 1842.

Every physician who has much practical acquaintance with disease, will have observed that there are great differences of character among the cases to which he finds it convenient, in accordance with the custom of medical men, to give the general name of *croup*. He finds that a certain portion of these cases—and by far the larger portion—yield readily to the means which he employs, and very often to the ordinary domestic remedies of mothers and nurses. He has indeed reason to believe that a considerable number of them would spontaneously subside if left to themselves. On the other hand, he finds that there are some cases, fortunately but few in proportion to the whole, which exhibit throughout their course, a character of obstinacy that bids defiance to treatment, and which, with few exceptions, pass on to a fatal termination uninfluenced by any remedies he can employ.

Different views may be taken of the nature of these cases. It is believed by some that the former are not, for the most part, essentially different from the latter; that the difference is more in degree than in kind, or that the difference in the severity and result depends in difference of management; that the favorable character and course of the former, are merely owing to early and judicious treatment, and the fatal event of the latter to the inefficient or too tardy application of remedies. A long, and I trust a faithful examination of this disease has, however, satisfied me that this opinion is not correct. I have been led to believe that there is an original and essential difference in these cases; that those of the first kind are pathologically different from the second; that the former, even if they terminate fatally, which happens in some rare instances, do not terminate in the same way, or at least do not exhibit the same morbid conditions; and that no variety or deficiency of treatment will cause a case of the one kind to assume the character of the other.

I do not, however, mean to imply, that all the cases to which I refer, are capable of being classed under two varieties. Among those which I have characterized as the more mild and tractable sort, we still find great differences in the mode of attack, course, and mode of termination, and also in the degree in which they appear to be influenced by remedies. The object of this paper is to endeavor to contribute something towards determining the nature and extent of the distinctions referred to. With this view I have made an examination of all the cases of croup of every kind which have occurred during the last twelve and a half years, in my own practice, and of this examination I now submit the results. Upon certain points relating to the severer form of the disease, I have included the examination of a number of other cases, extending over a period of twenty-five years, witnessed partly in my own practice, partly at dissections, and partly in consultations.

It should be first observed, that, in noting cases in order to this inquiry I have set down as croup, all those which in the common language of the profession are included under this name—viz., all those which, at any stage of their progress, present a fair question of diagnosis; all those in which is heard that shrill, sharp, ringing cough, which is regarded as the cough of croup, accompanied by a distinct embarrassment of respiration, however slight, and by some affection of the voice. It follows of course, that many very slight cases must have been included among those on which these remarks are founded—cases which yielded or subsided almost at once. Yet it is right that these should form part of the materials of our examination. When we are in search of means of diagnosis, our attention should be directed to all those cases which have, at any period of their progress, exhibited symptoms that give rise to a well grounded suspicion of their character. Although many cases which excite the apprehension of severe croup on their first attack, pass away very readily, and by their result show themselves to have been of very moderate severity; yet, on the other hand, it is to be recollected, that many cases, which at last terminate fatally, do not at their beginning, exhibit symptoms at all more severe, or excite apprehensions at all more serious, than those which have so readily subsided.

Of the cases to which this inquiry relates, that occurred during the period extending from Jan. 1830, to July, 1842, the number is 131. For the convenience of examination, these may be divided into four classes. I do not intend by this arrangement to express the opinion that they constitute four distinct diseases. I would not even be understood to assert positively, with our present amount of knowledge, that they are not different manifestations of the same disease. The purpose now is to speak of them as groups of cases distinguished by certain differences in their symptoms and course, which may or may not be connected with an essential difference in their nature. These classes may be designated, with a view to their probable character and for the purpose of referring to them more intelligibly, by the terms membranous, inflammatory, spasmodic, and catarrhal. Of the whole number there were:—

| | Cases. | Deaths. |
|---|---|---|
| Of Membranous Croup | 22 | 19 |
| Inflammatory " | 18 | 0 |
| Spasmodic " | 35 | 0 |
| Catarrhal " | 56 | 0 |
| | 131 | 19 |

In the first class are included those cases in which there is reason to believe that a false membrane has been actually formed, lining the larynx and trachea.

In the second class, those cases in which the symptoms are for the most part, of the same character as in the first, but in which there is reason to believe that no membrane has been formed. The grounds for the opinion formed of the nature of these two classes will be stated subsequently.

The terms applied to the third and fourth classes, require no particular explanation.

The symptoms on which we depend for the diagonis of croup, relate to the cough, the voice, and the respiration.

In the early stage of the first form of croup, the cough is by no means peculiar. In the advanced, it assumes a somewhat different character. In the early period it is sharp, shrill, ringing; it does not vary from that which we hear in the other forms, except perhaps that in some of the less formidable cases it is much louder and more violent at the beginning, than it is in those which prove ultimately more alarming. In the latter period it becomes less loud and ringing, but is equally sharp—it often becomes almost inaudible, bearing the same relation to a common cough, that a whisper does to the common voice. The cough, then, affords no certain means of distinguishing this form of croup at that period of it in which the diagonis would be most valuable.

Of the state of the voice, nearly the same remark may be made. In the advanced stage of a case it is sufficiently characteristic. It becomes a sharp, and almost inaudible whisper. But early in the disease it is not always affected at all; and, if it be, cannot with certainty be distinguished from the hoarse voice of common catarrh.

The condition of the respiration affords us far more important information. In the early period of the disease, however, when we most need means of diagonis, it is not a symptom which always attracts attention, even from the physician; much less from others who are around the patient. The common description of the breathing in croup, does not apply well to the beginning of the membranous variety. It seems rather taken from cases of a less dangerous kind, in which the breathing is from the first, loud harsh, suffocative ; attended with great efforts, and much loud coughing; creating great alarm, and calling at once for efficient means of relief. But the breathing in membranous croup does not excite attention in the very commencement of the disease. It is comparatively quiet and unobtrusive. Its true character is not at once to be detected, but only by a careful and accurate observation. The patient has not the ordinary aspect of difficult breathing; in fact, the breathing is not difficult at the very first. He probably experiences no distress. There is no real deficiency in the performance of the function, and no obvious embarrassment. There is only a little effort in drawing in the air, and a little more force exercised in its expulsion, whilst the amount of air admitted and expelled is fully equal to the necessities of life. This perhaps would not be noticed on a casual glance at the patient, but will be at once perceived on attending to the muscular movements subservient to the function, which are—to use an expressive French term—somewhat exalted. It is indicated very soon, also, by a slight dilatation of the nostrils, and a little whiz or buzz accompanying the passage of air through the rima glottidis. This sound is distinguished either by placing the ear near the mouth of the patient, or by applying the stethescope on the back of the neck, or directly upon the upper part of the larynx.

This at its very beginning is the essential respiration of membranous croup, and it affords far more aid in diagnosis than either the cough or the voice. It is not, however, always found as pure as has been described. It is often mingled with, and obscured by other sounds. Thus the disease is often attended by paroxysms of irregular and spasmodic breathing, accompanied by violent muscular efforts and great distress, and of course producing other and more obvious sounds than those described. There is often also present in the air passages, either above or below the glottis, a quantity of mucus, giving rise to a constant or occasional rattling, which seems to mask the proper sound of croup. These adventitious sounds, being also as frequently heard in the other forms of croup, are therefore of no service in diagnosis. Generally there are intervals of relief from these superadded symptoms, especially immediately after vomiting or bleeding, but the essential breathing of the disease will be found to be unchanged and unmitigated in these intervals of ease; although the apparent relief may be so considerable as to give rise to strong, but fallacious hopes of recovery.

We occasionally hear, in cases of considerable enlargement of the tonsils, a kind of breathing which closely resembles the early breathing of croup. Usually in such patients the respiration is loud, sonorous, unequal, and irregular, but in a few it is quiet, steady, with a muscular effort occasioned by a mechanical obstruction like that in croup. The distinction between them can, however, be readily made, by attending carefully to the seat of the obstruction, which is above the rima glottidis in the one case, and at it in the other; by the sound of the cough and voice, which are not croupy, and by the fact that the obstruction varies in degree and sometimes vanishes, with change of position.

I have endeavored to describe the respiration as it exists in its slightest appreciable degree, at the earliest period of its manifestation. As the disease advances, it becomes very strongly marked, whilst the condition on which its peculiar character depends, viz., a mechanical narrowing of the orifice through which the air passes, becomes much more obvious.

The muscular effort, in the latter stage, becomes very strong, both in inspiration aud expiration. During inspiration, whilst all the muscles concerned in it are in the highest state of activity, the mechanical impediment against which they act, is often strikingly displayed by the falling in of the soft parts about the neck and clavicles, at the epigastrium, and between and along the lower edge of the ribs—the air not passing in through the narrowed opening of the glottis so rapidly as the dilation of the chest by the increased muscular effort would render necessary. The expiration is chiefly characterized by the amount of force employed to expel the air. In health the expiration is easy, and accompanied by little effort. Where there is an unusual obstruction, the mere tendency to collapse of the lungs would be sufficient for the expulsion of the air, as we see in the dead body; so that the walls of the chest have merely to follow up this contraction, without adding to its force by any muscular effort. But in croup, this is not enough; and we often find that the air is blown out forcibly against the mechanical resistance occasioned by the disease. We find the same strong contraction of the muscles concerned, especially of the abdominal muscles, which is observed when air is blown out forcibly through a narrow passage.

This is the proper breathing of croup; becoming more and more intense as the disease approaches its termination, till the whole life of the individual seems, as it were, to concentrate itself in this one effort. The patient in this extreme condition seeks, by a multitude of changes of place and position, to find some alleviation of his agony; the cough, and with it the voice, have become nearly extinct; and his inarticulate appeals and beseeching looks for relief to those from whom he is accustomed to look for it, constitute one of the most touching scenes which we are called upon to witness in the practice of medicine. Happily the extreme suffering usually, though not always, subsides towards the close of life, and death takes place at last with comparative ease.

In the advanced stage of croup, the breathing is often modified by circumstances other than the mere mechanical obstruction at the upper part of the larynx. After a certain period the false membrane is in some places separated from its adhesion to the mucous surface, by the secretion of pus. The passage of air to and fro, and the efforts of coughing detach it partially from its adhesion, and break it up more or less into shreds, which however still adhere at one of their ends. These rugged portions of membrane, mingled with the pus, move up and down the air passages, causing some variety in the sounds and also in the actual difficulty of breathing. Death is sometimes very suddenly produced by a collection of this material into a mass which becomes impacted in, and thus plugs up, either the upper or lower part of the larynx. This at least, from the state in which the parts are found on dissection, would appear to be the mode in which death takes place.

The respiration may also be modified in croup from a congestion or inflammation of the lungs, which occasionally supervenes. The embarrassment of respiration has also sometimes appeared to be increased by an accumulation of air in the lungs, which arises from a deficient balance between inspiration and expiration. Owing to the greater ease with which we can make extraordinary and continued effort of inspiration than we can of expiration, a greater quantity is admitted than can be readily expelled, before the suffocative feeling of the patient impels him to a new effort for relief.

But although there may be a combination of the respiration of this disease with that produced by other affections of the throat or lungs, yet the respiration of croup is in its nature and character essentially distinct from them. In them the difficulty of breathing, and the unusual muscular effort, may arise from a variety of causes, producing great varieties in the modes of dyspnœa; in croup, the one essential condition is the mechanically contracted state of the passage through which the air passes, and all the peculiarities of the dyspnœa proceed from this condition. In one particular, the breathing of asthma resembles that of croup, viz., in the intensity of the effort by which the current of air is made to move in both directions against a mechanical resistance; but the point of the resistance, and consequently the other circumstances of the function, prevent the resemblance from extending to other points.

The *first* form of croup, then, is distinguished by the cough, the voice, and by a peculiarity of the respiration which I have attempted to describe, and which, for the sake of distinguishing it in this essay, may be called *intense.*

In the cases of the inflammatory croup, which constitute the *second* form of the disease, the condition of the voice, cough and breathing, are precisely the same as in the cases of the first class. There is no certain way by which, so far as these symptoms are concerned, cases of the one kind are to be distinguished from those of the other. The cases enumerated among the second class, were of all degrees of severity, but not one of them was fatal. Cases, however, of croup which terminated fatally, and in which no membrane was found on dissection, are recorded upon the best authority. To those we shall have occasion to advert hereafter. In addition to the symptoms proceeding from the character of the cough, voice, and respiration, I have noted, in a few examples of this form of the disease, a tenderness of the larynx on pressure.

As cases of this class are then usually favorable in their termination, whilst those of the first are usually fatal, the diagnosis between them, in the early stages especially, becomes of very great importance, both as regards prognosis and treatment. Of the means by which this distinction may probably be made, and of the grounds for believing these two to be essentially distinct diseases, and not different states or conditions of the same disease, I shall take occasion to speak, after considering the other two classes which have been enumerated.

The *third* includes certain cases, generally designated as *spasmodic croup,* and sometimes as *spasmodic asthma.* The attack is always sudden, and usually occurs after the subject has been, for some time, asleep. Very often it occurs in the evening, during the first sleep of the child, before its parents have retired to bed; but perhaps as frequently at a later hour of the night, or very early in the morning. The patient wakes in great distress for breath. His inspiration is attended with great effort; it is loud, ringing, shrill, somewhat resembling the hooping inspiration of hooping cough, but louder and more sonorous. The expiration is comparatively quiet and easy. The voice at the same time, is hoarse and broken, and there is a loud, hoarse barking cough, which closely resembles that of the preceding kinds, and indeed alone, would not serve as a mark of distinction from them. These cases seem occasionally to arise from indigestion; but more frequently we can trace their occurrence to cold, especially as they have been often preceded for a few days by symptoms of catarrh. When left to themselves, they will usually subside spontaneously, but from their suddenness and violence, they cause great alarm, and call for immediate assistance. They rarely fail to yield to an emetic or venesection, leaving behind them for a longer or shorter period, rarely for more than twenty-four hours, some hoarseness and some degree of the croupy sound of the cough, with a little huskiness or stiffness of breathing. At no period is there any proper *intensity* of respiration.

These cases, from their suddenness, the time of the attack, the great violence of the first symptoms, and the consequent alarm which they create, produce a stronger impression on the minds of common observers, and even of many practitioners, than those of the other kinds. This mode of attack is most closely associated in their minds with the term croup; and it is regarded as tending, if not checked, to terminate in the same state of things with cases of the first class. So far as the cases before us are concerned, however, this never happens, and of the whole number included under this examination, no one proved fatal.

The *fourth* class includes cases not falling under either of the above, and yet frequently presenting a very close resemblance to them. The subjects usually exhibit at first the symptoms of common catarrh. After a few days, the voice becomes hoarse; the cough becomes croupy, and there is tightness, oppression, and some approach to the croupy sound of respiration; there is, however, no intense or exalted action of the respiratory muscles, and no indication of that mechanical impediment to the current of air which exists at the rima glottidis in the two first forms of the disease. Still the resemblance is quite close enough to cases of the same forms, in their earlier stage, to occasion some anxiety, and there is also sometimes a sudden attack of dyspnœa, with loud, shrill and sonorous breathing, which imitates the symptoms of the third form, and is perhaps to be regarded as an attack of the same kind.

The cases of this form yield gradually, the croupy character wearing off in a few days, and leaving behind simply catarrhal symptoms. I suppose them, from the mode in which they come and go off, to be properly a catarrhal inflammation of the mucous membrane covering the organs of voice. We frequently observe that the catarrhal affection of the same membrane which occurs in the first stage of measles, is accompanied by the same croupy symptoms as those which have been now described—going off with the other catarrhal symptoms. In a few instances the attacks of this form of croup have terminated in severe bronchitis, or in inflammation of the lungs themselves. But among the 56 cases included above, there was no one fatal.

Having thus described these several forms of this disease, and stated in general what seemed to be their nature, the question now arises as to the justice of the distinction which has thus been assumed to exist. Is there any sufficient ground for such a distinction? Are these different cases different diseases? Are not the favorable ones, which constitute so large a proportion of the whole number, similar in their nature to the more severe; but either of less severity in their origin, or else modified and controlled in their course, by the influence of treatment? These questions, it is obviously of great importance, to the prognosis and treatment of the cases in question, to be able to answer correctly. If we can, with regard to a large proportion of them, confidently predict from the outset a favorable issue, the practitioner and the friends will be saved much unnecessary anxiety, and the patient many annoying and debilitating remedies.

I proceed, therefore, to state the grounds for a belief that the first form of croup is a disease essentially distinct from all the others, and that it depends on a peculiar pathological condition to which they have no tendency. Whether there be any equally marked distinction between the other forms, it is not of the same practical importance to determine; and as we have no sufficient materials for a satisfactory inquiry into this question, our attention will be confined to the evidence for the distinct character of the first form.

Every physician is familiar with an affection of the throat, both in adults and children, consisting in an inflammation of the mucous membrane, of that peculiar character which produces the effusion of a layer of coagulable lymph, or false membrane. The connection of this affection of the throat with croup, was long since pointed out; and it is well known to practitioners among us, that this complaint, known familiarly, though inaccurately, under the name of "ulcerated sore throat," often accompanies or is followed by croup, and that croup thus connected is peculiarly fatal in its character. This circumstance in the history of croup, was many years since strongly impressed upon my mind by an eminent practitioner in this neighborhood.* I was in consequence led, in all cases of croup, subsequently to this period, to make a careful examination of the fauces, with the view of determining exactly the extent to which this visible affection of the throat was connected with the more important disease.

Two causes prevent the completeness of these observations. We are very apt, in making record of cases, especially of those which appear of a slight degree of severity, to omit the *noting* of negative facts, even when they have been actually the objects of attention. Hence, although I have very rarely failed to examine the fauces in any case of supposed croup, I have often in the lighter cases, and sometimes in the severer, failed to note their condition. The second cause of incompleteness is the impossibility in some patients, from their terror and consequent resistance, of getting such a view of the parts as would authorize us to pronounce decidedly what their state is. Notwithstanding these circumstances, the state of the throat has been noticed and recorded in a sufficient nnmber of cases, to afford very fair materials for inference.

With a view to this examination, I may include a considerable number of other cases, besides those which constitute the particular subjects of inquiry in this paper, which have been noticed at other times, or in the practice of my friends. Including these cases with the 22 above referred to, I have memoranda, more or less complete, of 39 cases of what I have denominated membranous croup. The state of the fauces was observed and noted in 33, and of these, in 32 a false membrane was present; most frequently, and sometimes only on the tonsils, sometimes on other parts also, as the palate, uvula, and pharynx. In one case, no such membrane was present; but it was found to exist in the larynx after death. In three of

* Dr. William J. Walker.

these 33 cases, recovery took place; all the others were fatal. In 14, an examination was made after death, and the usual appearances were found to exist in all of them.

On the other hand, I have memoranda of 109 cases of what I have classed as the other forms of croup, and of these the state of the tonsils and fauces was noted in 45. In no one was there such a condition of the parts as was found to exist in the membranous form. In three cases there was indeed, a thin, slight exudation on the tonsils, of the color and appearance of starch, like that wihch is sometimes seen on the edge and surface of the tongue. This I apprehend to be a formation of an entirely different nature from that which exists in the other class of cases. Of the 45, 12 were of the second, 11 of the third, and 22 of the fourth class.

From this statement, it seems probable that the appearance of a false membrane upon the tonsils or other visible part of the throat, in a case of croup, may be regarded as a pretty certain diagnostic sign that it is the membranous form of the disease; and its absence as a pretty certain indication that it is one of the other forms. Still there will be exceptions. There will be cases in which the membrane is formed in the larynx, although it has not appeared in the throat; and there may be those in which a membrane exists in the throat, unaccompanied by a similar condition of the air passages. Of the former, I have recorded one example; of the latter, none. How freqnent such exceptions will be, must be determined by more extensive observation. If they are not more frequent than they have been among the cases here recorded, the observation of this symptom will afford a sufficient safe guide, since of 75 cases in which it was looked for, and the result noticed, it failed as a diagnostic sign in but a single instance.

The question now presents itself, what are the grounds for believing that the two forms of the disease which I have distinguished as membranous and inflammatory, are not the same in different degrees or in different stages? and may not pass one into the other? The grounds are—

1. The very great preponderance of fatal results in the membranous croup, and a similar preponderance of recoveries in the inflammatory, and the evidence which exists that in the few cases of recovery from the former, the membrane has been formed, and in the few cases on record of death from the latter, that a membrane has not been formed—afford strong reason for believing that the diseases are essentially different.

2. The formation of a false membrane does not seem to require either an advanced stage or a very intense degree of the inflammation from which it proceeds. It is rather the result of a peculiarity in the kind of inflammation, than of any period or degree of it. It appears to be a very early product of the inflammation, if it be not indeed almost contemporaneous with it. It resembles in this respect the similar effusion taking plaçe on the serous membranes, which in them occurs very early, and has ever been supposed to be the first act of inflammation. In the common inflammation of the tonsils which is accompanied by this symptom, a layer of lymph is observed to be effused over the surface of the part as soon as any signs of disease exist.

3. The circumstances attending recovery from simple inflammatory croup differ materially from those which accompany recovery from membranous croup. In the former, the amendment is rapid and speedily completed.

There is left behind only a moderate soreness of the larynx, and, in the worst case, some hoarseness. There is at no time any copious or solid expectoration. In the latter, recovery is slow, unequal, and accompanied by phenomena which must necessarily attend the separation of the membrane, and the process through which the diseased mucous surface must go in order to its restoration to a healthy condition. The natural cure of the disease takes place by the occurrence of the suppurative inflammation upon the diseased surface, by which the false membrane is thrown off, and the mucous membrane then gradually returns to its natural state. In examinations after death, we usually find that this process has begun in the trachea, the membrane being there separated and often broken up into shreds, whilst the inflamed surface is covered by a layer of pus. Above, in the upper part of the larynx, around the glottis, the false membrane usually remains closely adherent. It is obvious that recovery might always take place, could the parts be spared long enough from their functions to go through the necessary steps—and it is also obvious when it does take place, that it must be accompanied by a copious expectoration of pus, and of the membrane either in pieces, if firm enough, or else broken up and partially dissolved by the pus. Now these appearances do not accompany recovery from even the severest cases of the inflammatory croup, whilst they do accompany recovery from well-marked cases of the membranous form.

Of the three cases of membranous croup which are noted as having recovered, there are but two of which I have such an account as would justify me in presenting them as fair examples of the processes through which the parts pass in recovery. These were both of the most decided character, and had arrived at that stage of the disease in which we expect a fatal event to occur almost from hour to hour. In the first of them, six days elapsed before any sensible mitigation of the symptoms, and even then the progress to recovery was very slow, and apparently doubtful. Improvement was attended by a copious muco purulent expectoration, in which it is true no large pieces of membrane were ever detected, but of such a consistence and appearance as would favor the belief that the membrane had escaped in a comminuted or partially dissolved state. After the probable removal of the membrane, there was for some days a bloody expectoration, the voice did not return, and it was indeed many weeks before it resumed its natural tone.

In the second case, a considerable portion of the membrane was spit up in a tubular form, after a violent fit of suffocative cough, and this was followed by the rejection of smaller pieces, mixed with a muco-purulent, at first, and then a bloody expectoration. There continued an entire loss of voice for more than a week, and for at least ten weeks after recovery, it had not regained its natural tones.

The contrast is very striking between the protracted character of these recoveries, and the speedy return to health of all those who labored only under the other forms of the disease, however severe.

The observations to which the preceding remarks relate, were all made in this city and its immediate neighborhood; how far they correspond to the disease as it appears in other places, must be left to others to judge. So far as they go, they appear to me to justify the following conclusions:—

1. That the only form of croup attended with any considerable danger to life, is that which is distinguished by the presence of a false membrane in the air passages.

2. That the existence of this membrane in the air passages is in a very large proportion of instances, indicated by the existence of a similar membrane in the throat.

3. That this affection differs not in stage or degree, but in kind, from all the other cases which are commonly known by the same name, and that the latter have no tendency to become converted into or to terminate in the former.

As my intention has not been to write a complete history of croup, I have omitted all such notices of the symptoms, cause, morbid anatomy, &c., of the disease as have no direct bearing on that point in its character which it was my desire to illustrate. It may not be amiss, however, to record, in connection with this paper, a few circumstances with regard to its history, which have been incidentally determined from an examination of the cases before us.

Croup is often regarded as a disease which attacks suddenly and violently. This is only true of the milder forms. Genuine or membranous croup is commonly rather gradual in its approach, and consequently often insidious. It supervenes often on the common sore throat of children; and in such cases, though its development is frequently rapid and apparently sudden, yet a careful examination of the past history of such a case will generally satisfy us, that although it may have had a sudden outbreak of violence at the time it was supposed to begin, yet that it had really been coming on for several days. Of 30 cases in which I have had an opportunity of determining the mode of attack, in only two could it in any proper case be called sudden, although in many, the attention of friends was called to it quite unexpectedly, by a rapid increase in the violence of the symptoms. A sudden and violent attack is, therefore, to be regarded as affording a favorable indication of the character of the case in which it occurs. The unexpected manner in which croup sometimes steals upon the common sore throat of children, should lead always to the careful inspection and watching of such cases. It is true that but a very small proportion of them do terminate in this way; but as it is the only considerable source of danger, and the only way in which they are likely to have a fatal termination, the possibility of such a course of things should not be overlooked. No case of this kind can be regarded as entirely safe from such a result. The danger is even not confined to childhood. Two of the above-named cases of fatal croup, occurred in females of 12 years of age, in which it had supervened on this affection of the throat.

The membranous croup also sometimes occurs as a sequel to the affection of the throat in scarlatina. The most common primary affection of the throat in this disease, is of the same kind with that denominated the ulcerated sore throat, viz., an inflammation, with an effusion of false membrane upon the parts inflamed. When croup supervenes upon this, the case is usually very rapid, and invariably fatal. Of the cases above enumerated, two were of this character. A third occurred to me, not enumerated among them, in which there was no symptom of croup during life, the patient apparently dying from affection of the brain, but in which the usual appearances of croup were found after death. The subject of this was a young man 17 years of age. These cases all occurred between eight and ten years since. None have been observed during the more recent periods of the prevalence of scarlatina.

Croup varies considerable in its duration; I mean its duration after its characteristic symptoms are fairly developed and there is reason to believe that the membrane is formed. Of 23 cases,

| | | | |
|---|---|---|---|
| 1 | continued | 1 day | from distinct croupy symptoms. |
| 6 | " | 2 to 2½ | |
| 9 | " | 3 to 3½ | |
| 3 | " | 4 | |
| 1 | " | 5 | |
| 1 | " | 9 | |
| 1 | " | 11 | |
| 1 | " | 19 | |

Nineteen cases, or more than three-fourths, therefore, were of four days' duration, or less. * * * * * * * * *

II.—*Treatment of Croup*. Read before the Boston Society for Medical Improvement, by John Ware, M. D., Nov. 11, 1844.

The history of the case of croup reported at the last meeting,* which I had an opportunity of witnessing during its progress, has confirmed me in an opinion I have, for some time, been disposed to entertain, that the methods of treating this disease in common use, require a a careful re-consideration. This opinion is connected with, or perhaps has proceeded from, certain views concerning the distinctive character of various forms of disease which ordinarily are included under this one common appellation, and which I have formerly communicated to the Society. It is not too much to say, that the received mode of treating these cases, which, so far as I know, is very much the same for all their varieties, has come down to us by a sort of tradition, from our predecessors. It is true that in single cases and by particular individuals, there have been occasional variations from the established practice; still in the main, emetics and bleeding, blisters and calomel, have been the principal remedies. The depleting, reducing and perturbating methods is that on which dependence has been chiefly placed.

That this treatment may be applicable to a very considerable proportion of the cases which pass under the common denomination of croup, I am not prepared to deny. Those which in a preceding communication have been classed as inflammatory, spasmodic, and catarrhal, certainly recover under its influence, and apparently with greater speed than if left entirely to the resources of nature. So far as my experience has gone, however, it has appeared to produce no impression upon those in which there is satisfactory evidence that a membrane has been formed.

These cases, I should repeat the opinion expressed in the paper just referred to, are essentially of a distinct nature from the others, and constitute but a small proportion of those which are usually regarded as croup.

* This was the case of a child with membranous croup, communicated by my brother, Dr. Charles Ware, of this city, in which the anodyne treatment was mainly employed, and in which the membrane was separated and thrown off. Every thing promised favorably for recovery so far as croup was concerned, but the patient died ultimately by the rapid supervention of inflammation of the lungs.

They are not aggravated cases of the same kind as the others—cases which have gone on to an ulterior stage of disease—but in their origin and conception different. The inflammation which is essential to them is peculiar in its character; the effusion of false membrane is not the result of an advanced stage of it, but is one of its early results—is perhaps the first visible act of its existence; as there is much reason to believe that it is of serous membranes. It has been common to describe the stage of effusion in croup, as preceded by one of longer or shorter duration—a formative stage. If I am right in the views taken of the character of the disease, this distinction is made by making up its history from different sets of cases—going to one for the history of the first stage, and to another for the history of the second. The same confusion of diagonis has given also an apparent success to means used for treatment. Where all the different cases which have been referred to, are grouped together as examples of the same disease in different stages or degrees, the proportion of recoveries will not appear discouragingly small. If we were to class together, as cases of consumption, all those in which there was cough and expectoration, as is done by those who profess to cure this malady, we should have no reason to be disheartened with regard to its curability; and, in the same way, so long as we class all cases together as croup, which have a croupy cough and some difficulty of breathing, the amount of mortality will not be greater than in other acute diseases of children. A more accurate diagnosis will, I am convinced, put an end to our complacency on this point. Membranous croup unquestionably does sometimes come to a favorable termination; but recovery is comparatively so rare, it forms so much the exception, that, admitting the distinctive character of the disease, it is difficult to conceive that the treatment has any thing to do with the recovery. Where, under any given method of treatment, but one case out of six or eight recovers, one must be very sanguine indeed to attribute much influence upon the result of the remedies.

The question then properly arises—if the mode of treating croup commonly adopted does no good, are we sure that it does no hurt? This is a question we are far too unwilling to put to ourselves. What will happen if nothing be done? This should always be the first thought of the physician, in each individual case. Till he knows this, he cannot know with certainty what effect his treatment has; and just in proportion to the amount of his knowledge of the natural history of disease, and of the time and mode of its natural termination in recovery or death, will be his power of judging of the influence of treatment upon the result.

Now when we examine the cases of recovery of membranous croup which actually take place, and compare them with the condition of the parts in those which are examined after death, we find very clear evidences of a tendency in the disease to go through a certain course of changes which will terminate in health. The false membrane is effused, and, at the same time, the mucous membrane is thickened and congested. After a time, a process of suppuration is established upon the surface of the mucous membrane, underneath the false membrane, which of course separates the latter from the former, so that it lies loosely upon it, whilst between them is a layer of pus. If the membrane thus thrown off be thick and strong, it is expectorated in distinct pieces, sometimes of a considerable size; if it be thin and less firm, it is either converted partially into pus, or else is broken up into smaller shreds and mixed with the pus so as not to be distinguished from it, except by very careful examination, and thus it is all gradually thrown up. The diseased membrane does not free itself from the false membrane over its whole surface at once. Those portions from which the false membrane has separated, are left in an inflamed and irritable state—the expectorated membrane and pus are often tinged with blood, probably from the fact that by the violent effort of coughing some portions are torn off from the mucous surface before the purulent process had effected a complete separation. The cough, then, with more or less expectoration, and a hoarseness, in some cases amounting to an incapacity for speaking except in a whisper, continue for some time—the affection of the voice for several weeks. The parts are at length, however, perfectly restored.

In cases which prove fatal, we find evidences that the same succession of changes is taking place; that an effort has been making to bring about the same result. It is in fact from the examination of the progress which has been made in fatal cases, that we are enabled to judge what is the exact condition of the parts, and what the processes through which they go, in those which recover. Thus in some portions of the organ affected, we find the false membrane very closely adhering to the mucous, whilst the latter is reddened and thickened. This especially occurs at the top of the larynx. Lower down the false membrane is more or less extensively loosened from its adhesion—usually irregularly so—whilst a layer of pus lies between it and the mucous membrane. In some places the effused coat has been entirely separated, and has been either spit up, or else is found loose, enveloped in pus, in some part of the passage; whilst the surface to which it adhered is red, swollen and besmeared with pus. Thus we trace every where distinctly the existence of a process, the tendency of which is obviously to bring about recovery; but death has taken place before it has been comple'ed. It takes place in different steps of the process. Sometimes quite early, before any separation has taken place, the patient apparently dying from the diminished aperture of the air passages from spasm and inflammation. Sometimes later, when the separation has taken place below, but not at the top of the larynx. At other times the membrane separates in considerable quantities, becomes collected into considerable masses, and produces suffocation by being wedged in at the bifurcation of the trachea or at the very top of the larynx. There are other cases in which recovery is also obviously taking place from croup, but in which death occurs from the supervening of secondary disease in the lungs.

Croup, when once established, can then only be recovered from, by going through with this regular course of changes. These are essential to it. When once this process has begun; when the false membrane has been fairly effused, the parts can no more recover without them than the eruption of small-pox can be cut short in its progress. A rational method of treatment, then, is that which will promote the necessary changes. And what do we need? 1. To prolong life, to prevent suffocation, in order to give time for the required process to be completed by the efforts of the organs themselves; and 2. To use means which will promote and hasten this process—which will aid the system in the work which she is aiming to perform.

Now are the usual means likely to answer these purposes? Have they answered these purposes? That emetics and bleeding sometimes relieve violent turns of dyspnœa, must be admitted; yet that they actually prevent suffocation in many cases, admits of very great doubt. But do they contribute at all to those changes upon which alone we can depend for actual recovery? There is no evidence that they do; whilst on the contrary there is reason to fear that they may interfere with them, may retard them, may prevent them. If, then, these remedies be at best of doubtful efficacy, is it not right, in so formidable a disease, to make the trial whether other measures may not be more successful? At any rate, if other means are not more successful, they may at least be less tormenting to the patient, and inflict a less amount of unnecessary suffering.

It is to be remarked of the case which has suggested these observations, that the subject of it rejected all remedies, so that it was in fact a case left very much to the resources of nature. Still, so far as the morbid condition in which croup consists is concerned, recovery was very fairly taking place, and would have been complete, except for the occurrence of a secondary affection. I may say also of the very few cases which I have seen completely recover by the expectoration of the membrane, that they were not the subjects of very active perturbating treatment, especially after the first stages had gone by, but were left a good deal to palliatives—to mild, soothing applications. It would seem worth while, therefore, to make the attempt of treating the disease without the persevering use of the heroic remedies by which it has been ordinarily encountered; that we should —not perhaps leave the disease wholly to nature—but trust it at least to such remedies as will not interfere with that regular course by means of which nature is always attempting to give relief.

### III.—*Further Remarks on the Treatment of Croup.* Read before the Boston Society for Medical Improvement, Feb. 20, 1845.

Some remarks were presented to the Society a few months since, on the treatment of croup, including suggestions concerning the management of that form of the disease which is attended by the formation of a false membrane in the larynx and trachea. A case of the disease has since occurred to me, which seems to be worthy of notice in connection with those remarks.

The subject was a male, 5½ years of age; of pale and delicate aspect, and slender habit. He had not been perfectly well since an attack of scarlatina, two years ago; since then, he had been frequently liable to colds, with severe coughs. He had enlargement of the submaxillary glands and of the tonsils.

He was first seen on Sunday eve, Feb. 9, 1845. The account given by his parents was, that he had had a cough with a croupy sound—a sound with which they were familiar—for ten days past; but with it no trouble in breathing; that to day, however, his voice had become hoarse, and that he had several turns of hard, suffocative breathing. The cough and respiration were at this time distinctly those of croup, though at the time of the visit there was no distress. There was false membrane on the tonsils. He had taken an emetic of ipecacuanha and a dose of castor oil.

He was directed to take, once in three hours, 1½ grains of Dover's powder and half a grain of calomel—to sponge the neck frequently with warm water, and to apply to it this liniment—R. Olei oliv., ℥j.; aquæ potass., ʒij.; ung. hyd. fort., ʒj. M.

Feb. 10th.—The night had been easy, upon the whole, though there had been several turns of distress. During one of these he took two drachms of wine of ipecac., with free vomiting. The symptoms of membranous croup were perfectly well-marked, but there was no distress. The liniment was continued, a flax-seed poultice was applied to the neck, and the powders continued every two hours; to be suspended, however, if he became fully opiated.

During the day the voice became quite extinct; and the cough lost the loud and ringing sound which it presents in the early period of this disease. The breathing became more labored, and was accompanied by greater muscular effort, both in inspiration and expiration. Still he was not distressed, owing apparently to the influence of the opium. The air entered the lungs well. There was much sound of loose secretions in the larynx and trachea, but no expectoration, except of a little frothy mucus. It having been found difficult to keep the poultices in contact, the parents substituted boiled mullen leaves, which were assiduously applied. At the same time the patient was made constantly to inhale the vapor from a boiling decoction of the same plant, and this was persevered in uninterruptedly for several days.

It is not necessary to follow up a detailed history of the case. These measures were continued without change for several days, i. e., the poultice, the liniment, the inhalation, and the calomel and opium in sufficient quantities to keep him under a moderate narcotism.

On Feb. 12, Wednesday, there had been no distress of breathing; but its croupy character still continued; there had been no return of natural voice; but the sound of the cough had changed, and was like that of common catarrh—quite loose. Through Wednesday and Thursday there was much rattling of loose matter in the larynx and trachea, and it was coughed up in considerable quantities. Portions of the sputa were mixed with blood, and false membrane was detected in detached pieces enveloped in mucus and pus. One portion of it was of considerable size and distinctly tubular. The fits of coughing, especially when masses of false membrane were ejected, were suffocative, and the sputa were dislodged with difficulty. On Thursday there were still a large thick patch of false membrane on the tonsils. He was occasionally delirious. The pulse were about 120; the respiration varied from 12 to 20, and continued distinctly croupy, though without any distress. He was extremely prostrated.

On Saturday the respiration had lost the croupy character, but there was still a loose rattling sound in the air-passages, and the voice was unchanged. This day, for the first time, he manifested a little appetite, and his tongue became clean. He had continued occasionally to throw up pieces of false membrane.

On Monday, Feb. 17, he appeared perfectly well, except as to strength and voice. By considerable exertion he could make a slight approach to proper voice, but for the most part he spoke in a whisper.*

---

* This patient has had no return of the disease to the present time, March, 1850. His voice was not perfectly restored for many weeks.

The important point to determine in connection with this case, is how far recovery depended upon the treatment. The treatment consisted—

1. In the absence of all reducing, depleting, and disturbing remedies.

2. Keeping the patient under the full influence of opium, combined with calomel.

3. Constant external application of warmth and moisture, and of a mercurial liniment slightly stimulating.

4. Constant inhalation of watery vapor.

It is too much to say, that the recovery in this case was to be attributed, with any thing like certainty, to the mode of treatment employed. It may have been only one of those coincidences which so frequently mislead us in studying the effects of remedies. Still, as the expectoration of the false membrane has not been a very common occurrence under my observation, and recovery not universal, even where it has taken place, it will be at least useful to notice the circumstances which have accompanied a favorable case.

On the supposition that the successful result may have been connected in some degree with the treatment, I should be disposed to attribute it to the following circumstances:

1. To the absence of all such measures as tend to irritate the parts inflamed, and thus to interfere with the natural process of restoration—especially vomiting. That vomiting gives relief to the paroxysms of bad breathing in croup, will not be doubted; and so does it give temporary relief to the distress of an inflamed stomach. But relief of a symptom is not the cure of disease, and does not always tend to its cure. It is not in accordance with what we know of the effects of remedies in other inflamed parts, that concussion, motion, &c., should allay their inflamed condition. Vomiting relieves inflammation of some parts, and some kinds of inflammation; but in this case the parts inflamed are mechanically disturbed by the act, and it has, so far as we can judge, no probable influence upon that peculiar condition which constitutes the disease.

2. To the absence of all depressing and debilitating remedies—as bleeding, purging and vomiting, considered in their effects upon the system. Such means may be beneficial when we expect resolution of an inflammation. But where the successful issue of the disease depends upon its going through with a certain course of changes, as in croup, they are as likely to interfere with, as to promote them.

3. To the relief of the spasmodic contraction of the rima glottidis, which seems more or less to accompany its mechanical diminution by the effused membrane, and to aggravate very much the difficulty of breathing. It is probably upon the suspension of this spasmodic condition, that the temporary relief produced by vomiting chiefly depends, and especially vomiting by means of tobacco.

4. To the influence of external warmth and moisture in promoting the suppurative process, by which alone the false membrane can be safely separated.

5. To the constant inhalation of watery vapor. This may have promoted the separation of the false membrane by keeping it from becoming dried by the constant passage of air—and by rendering it pliable and soft, so as to be easily managed and expelled by the organs in the act of coughing.

These considerations lead to the belief that this method of treating croup is at least worthy of trial. But even should it not prove more successful, it is certainly vastly more comfortable than the ordinary method. The patient, whose case has been recorded, suffered very little after the first day, even before the extrication of the membrane. Indeed, taking the disease altogether, it was not attended by more distress than accompanies the average of the acute affections of children.

IV.—*Additional Remarks on the Treatment of Croup.* Read before the Suffolk District Medical Society, March, 1850.

Since the occurrence of the case described in the foregoing paper, I have had from various circumstances, fewer opportunities of witnessing cases of croup than in former years, and only five of this form of the disease have fallen under my notice. The three first of these were treated in the method pursued in the case above related.

The first case was that of a male, four years old, who was taken with membranous sore throat, accompanied by high constitutional irritation, Oct. 14, 1845. No croupy symptom occurred till Oct. 18, when they were manifested in a perfectly distinct manner. On the 20th and 21st, patches of false membrane with bloody sputa were raised—and one piece of four inches in length. The raising of the latter was accompanied by a severe and suffocative paroxysm of coughing. On the 22d he died, eight days from the commencement of the disease, and four from the access of croup. The suffering in this case was very considerable, but far less than I have been accustomed to witness in cases of croup treated according to the ordinary method.

The second was that of a female, four years of age, taken with croup on the 8th of Nov., 1845. No depleting or reducing remedies were employed. Patches of membrane, and one piece of considerable size, were brought up on the 10th and a few following days. She never suffered much, improved steadily, and on the 15th seemed well in all respects except the voice, so that on the 16th I did not see her. On the 17th, there was a return of all the croupy symptoms, including the appearance of lymph upon the tonsils, and she died on the night of the 19th, eleven days after the first seizure. During no part of the disease was the suffering from dyspnœa very intense for any continued period.

On dissection, the usual appearances were found, and in one lung the false membrane extended for some distance into the bronchi in the substance of the organ.

The third case was a female, six years of age, who was seized with the disease Oct. 31, 1837. The onset of the disease was gradual, yet quite distinct. Nov. 2d, the symptoms had become quite severe, and Nov. 3d, there was bloody expectoration, and pieces of membrane were spit up. Pieces of membrane continued to be found in the sputa for several days, and she was very comfortable and breathed with tolerable ease, yet never losing the distinct croupy sound of respiration and voice. On the 8th, she became rapidly worse, but without distress, and died on the 9th, quite easily, ten days from the first attack of the disease.

It will be admitted, I think, that these cases, especially the two last, exhibited certain differences from the common course of this disease, which indicated a favorable influence from difference of treatment.

In all of them the membrane was thrown up in considerable quantities.

In all of them the disease was attended by very much less distress than is usual in croup, and, in two, there was so decided a mitigation of symptoms following the separation of the membrane, as to lead to considerable hope of a favorable termination.

In two, at least, the disease was prolonged to at least twice its average duration under the usual treatment.

In the two other cases, to which reference was made, the same general course of treatment was followed, with the addition of the introduction of a sponge wet with a solution of the nitrate of silver, into the larynx. In each of these cases the application was made as early in the disease as I became satisfied of its distinct character. It was repeated morning and evening. It decidedly gave relief to the breathing soon after each application, and both cases ultimately recovered perfectly. For the suggestion and adoption of this valuable addition to our means of treating this formidable disease, we are indebted, as is well known, to the enterprise of Dr. Horace Green, of New York. The profession, I think, owe to him a large debt of gratitude, for the energy and perseverance manifested in the introduction of this remedy, and I am the more disposed to render this tribute to him because so many attempts have been made to detract from his merit in relation to it.

I am well satisfied from what I have now seen of this method of treating croup, as compared with that which has been followed for so many years, that it has the advantages which were pointed out in one of the preceding papers. It is a disease which I would treat without depletion—except perhaps by a few leeches—without vomiting, without purging, without blisters, without antimonials, ipecac., and all those other nauseous remedies which have been usually resorted to. I would trust to opiates, perhaps calomel, emollients, and the local application of the nitrate of silver.

I ought to add that many of my friends in the profession have informed me of cases in their practice, treated on these principles, which have recovered in a favorable manner. Among them I would refer to Dr. Fisher, Dr. Henry G. Clark, Dr. E. H. Clark, Dr. Buckingham, and my brother, (Dr. Charles Ware,) of this city, Dr. Cotting, of Roxbury, and Dr. Spooner, of Dorchester.

